# Risk of immune system and skin and subcutaneous tissue related adverse events associated with oxaliplatin combined with immune checkpoint inhibitors: a pharmacovigilance study

**DOI:** 10.3389/fphar.2024.1309540

**Published:** 2024-06-14

**Authors:** Chunhong Zhang, Furong Li, Ying Dai, Yifan Zeng, Xuben Yu, Dawei Shi

**Affiliations:** ^1^ Department of Pharmacy, First Affiliated Hospital of Wenzhou Medical University, Wenzhou City, China; ^2^ Computer Technology and Information Centre, First Affiliated Hospital of Wenzhou Medical University, Wenzhou City, China

**Keywords:** oxaliplatin, immune checkpoint inhibitors, type II hypersensitivity, rash, pharmacovigilance, signal mining

## Abstract

**Background:**

Few studies have analysed oxaliplatin-induced adverse events (ADEs) in the immune system and skin and subcutaneous tissues through pharmacovigilance. We used this approach to analyse the risk of such ADEs when oxaliplatin combined with immune checkpoint inhibitors (ICIs).

**Methods:**

We evaluated the association between oxaliplatin and ADEs in the immune system and skin and subcutaneous tissues using the reporting odd ratio (ROR) for mining the ADE report signals in the FDA Adverse Event Reporting System database. Risk factors were analyzed using a binary logistic regression analysis using the sex and age of the patients.

**Results:**

There were 40,474 reports of oxaliplatin as primary suspect drug or second suspect drug. The signal intensities of ADEs such as type II hypersensitivity, type I hypersensitivity, type III immune complex–mediated reaction, anaphylactoid shock and cytokine release syndrome were high in PTs classified by SOC as immune system disorders; in the PTs classified as skin and subcutaneous tissue disorders by SOC, the signal intensities of ADEs such as skin toxicity, skin reaction, rash maculo-papular and skin fissures were higher. In the risk assessment between the two groups, rash showed an increased risk in the oxaliplatin-ICI group, with an OR of 1.96. Nivolumab in combination with oxaliplatin had an OR of 2.196 and an adjusted OR of 2.231. Combined with pembrolizumab, OR was 2.762 and the adjusted OR was 2.678.

**Conclusion:**

Type II hypersensitivity shows a stronger pharmacovigilance signal. Oxaliplatin in combination with nivolumab or pembrolizumab has been shown to increase the risk of rash.

## 1 Introduction

Oxaliplatin is a third-generation platinum agent that is used in a wide variety of tumours, including pancreatic, biliary, gastroesophageal, and gynaecologic malignant tumours (Shao et al., 2010; Yu et al., 2021). Immune checkpoint inhibition therapy is a novel treatment regimen that is being progressively applied to treat a variety of solid tumours. Immune checkpoint inhibitors (ICIs) based on this regimen can prevent the immune escape of tumour cells by blocking the programmed cell death protein 1 (PD1)/programmed cell death-ligand 1 (PD-L1) pathway, thereby restoring the role of immune cells (Haanen et al., 2022; Johnson et al., 2020). Combinations of ICIs have emerged in addition to conventional chemotherapy drugs such as oxaliplatin, which have been combined in chemotherapy regimens for specific tumours (Galluzzi et al., 2020) and have achieved good clinical results in clinical practice.

Reports of oxaliplatin-induced hypersensitivity have been increasing yearly. Many patients have to stop treatment because of hypersensitivity reactions, with a discontinuation rate of about 21% (Yanai et al., 2012; Hewitt and Sun, 2006). Some studies have also suggested that the overall incidence of allergic reactions to oxaliplatin is between 2% and 25%, which seems to be independent of the type of tumour (Okayama et al., 2015; Shibata et al., 2009). The most common allergic reactions include pruritus, rash, urticaria, etc. (Rogers et al., 2019), and the median time of occurrence is usually in the first 4 cycles of chemotherapy treatment (Thomas et al., 2003). Meanwhile, immune-related adverse events (irAEs) have occurred during the use of ICIs, and the mechanism of such ADEs is still unclear (Zhu et al., 2021; Apalla et al., 2021). Among which skin tissue-related adverse events (ADEs) are the most common. And it is also reported that they often present within the first 2 cycles of treatment (i.e., within several weeks) (Villadolid and Amin, 2015; De Velasco et al., 2017).

In the real scenario, when patients experience the above-mentioned ADEs during OXA combined with ICIs treatment, it is difficult for clinicians to determine which drug dominates the occurrence of the ADE, thus making it difficult to accurately adjust the treatment plan reasonably. And whether such combination therapy increases oxaliplatin-induced skin allergic reactions or even life-threatening hypersensitivity reactions has not been reported.

The FDA Adverse Event Reporting System (FAERS) is a voluntary reporting system for ADEs. It can be used to evaluate the safety of drugs by collecting real-world ADEs (Takao et al., 2012). In this study, we used oxaliplatin reports in the FAERS database to analyze the oxaliplatin-related ADEs of the immune system, skin, and subcutaneous tissues by using signal data-mining methods and to assess the risk of such ADEs in the chemotherapy regimens of oxaliplatin combined with ICIs.

## 2 Materials and methods

### 2.1 Data source

The data used in the study are all from the FAERS database. Initially, data on adverse events recorded from the first quarter of 2013 to the first quarter of 2023 in the FAERS database were downloaded from the FDA website (US Food and Drug Administration, 2022). We built an original database that reintegrated the downloaded records using Oracle Database 11 g software and used SQL queries to retrieve relevant information.

The target drug of this study was oxaliplatin. We took a text-mining approach that searched for the drug in terms of its generic name and brand name (eloxatin). The target drug was set as the primary suspected drug (PS) or the secondary suspected drug (SS).

We followed the FDA’s recommendation to use the most recent case number to identify duplicate reports of the same patient that came from different reporting sources. Duplicate reports were also removed by matching age, sex, initial FDA date, and reporter country.

We also retrieved reports of the use of ICIs with oxaliplatin. This text-mining approach searched for the ICIs in terms of their generic and brand names: “nivolumab” and “opdivo,” “pembrolizumab” and “keytruda,” “atezolizumab” and “tecentriq,” “durvalumab” and “imfinzi,” “tremelimumab” and “imjudo,” “ipilimumab” and “yervoy,” “relatlimumab” and “opdualag,” “avelumab” and “bavencio,” “cemiplimab” and “libtayo,” “dostarlimab” and “jemperli.”

### 2.2 Definition of adverse events

ADE is described according to the preferred term (PT) and Systematic Organ Classification (SOC) in the Medical Dictionary for Regulatory Activities (MedDRA) 23.0 (Maintenance and Support Services Organization, 2022).

Immune System and skin and subcutaneous tissue-related ADEs (ISA-ADEs): ISA-ADEs were defined as adverse events related to immune system disorders and skin and subcutaneous tissue disorders. The SOC classification of immune system disorders was 10021428, and the SOC classification of skin and subcutaneous tissue disorders was 10040785.

### 2.3 Signal detection method

A disproportionality analysis was conducted by computing the reporting odds ratio (ROR) and the corresponding 95% confidence interval (CI) for the association between each ISA-ADE and oxaliplatin. The ROR was calculated as the ratio of the odds of reporting the ISA-ADE versus all other ADRs for a given drug compared to the reporting odds for all other drugs present in the FAERS (Almenoff et al., 2005). See details in Table 1. The following formula was used to calculate the ROR and 95% confidence interval (CI): ROR=(*a/c*)/(*b/d*), 95% CI = $e^{\ln\;\left(\text{ROR}\right)\pm 1.96\sqrt{\left(\frac{1}{a}+\frac{1}{b}+\frac{1}{c}+\frac{1}{d}\right)}}$ . An association was considered to be statistically significant if the lower limit of 95% CI was above 1.0 (Bate and Evans, 2009).

**TABLE 1:** Four-fold table.

Drugs	Target ADE (n)	Other ADEs (n)	Total
Target drug	a	b	a + b
Other drugs	c	d	c + d
Total	a + c	b + d	a + b + c + d

### 2.4 Data analysis

#### 2.4.1 ISA-ADE signal detection

Based on the ISA-ADE reports of oxaliplatin in the FAERS database, signal detection methods were used to mine the ISA-ADE signals.

#### 2.4.2 Risk assessment of ISA-ADE after oxaliplatin combined with ICIs

First, ADE reports of oxaliplatin were included in the ISA-ADE risk assessment study, and the reported patient population was divided into Group OXA, with oxaliplatin, and Group OXA-ICI, with oxaliplatin and ICI, depending on whether oxaliplatin was reported in combination with an ICI. The exclusion criteria were as follows: age, sex, and country (or region) of the report were taken as the judgement conditions; if any of the three items in the report had missing records, then the patients in the report were not included in the grouping study. The study excluded patients younger than 18 years of age.

Second, risk assessment was conducted between the two groups by using the results obtained from the previous signal-mining study on the ISA-ADEs, already defined as signals. The risk factors were analyzed by binary logistic regression analysis using the sex and age of the patients.

Microsoft™ Excel for Mac (16.72) and SPSS (Ver 25.0) were used for data processing and statistical computation.

## 3 Results

### 3.1 ISA-ADE signals of oxaliplatin

A total of 13,136,477 reports were included in the FAERS database. There were 40,474 reports of oxaliplatin as PS or SS based on established screening criteria.

All 34 ISA-ADE-related PTs were included in this study, and the reported frequencies and ROR values are detailed in Table 2. Among them, the signal intensity of ADEs such as type II hypersensitivity, type I hypersensitivity, type III immune complex–mediated reaction, anaphylactoid shock and cytokine release syndrome were all high, especially type II hypersensitivity (ROR = 208.099, 95% CI = 115.101, 376.236). In the PTs classified as skin and subcutaneous tissue disorders by SOC, the signal intensities of ADEs such as skin toxicity, skin reaction, rash maculo-papular and skin fissures were higher, especially skin toxicity (ROR = 44.555, 95% CI = 39.992, 49.639).

**TABLE 2:** The number of ISA-ADEs reports and the value of reports odds ratios.

SOC = Immune system disorders					SOC = Skin and subcutaneous tissue disorders				
PT	n	N	ROR (95% CI)		PT	n	N	ROR (95% CI)	
Hypersensitivity	989	118,091	2.776 (2.606, 2.958)		Rash	956	262,695	1.186 (1.112, 1.265)	
Anaphylactic reaction	386	30,258	4.212 (3.808, 4.659)		Pruritus	810	210,962	1.252 (1.168, 1.343)	
Cytokine release syndrome	306	7,429	13.998 (12.480, 15.702)		Skin toxicity	377	3,140	44.555 (39.992, 49.639)	
Anaphylactic shock	263	13,378	6.524 (5.773, 7.374)		Urticaria*	213	96,187	0.717 (0.626, 0.820)	
Drug hypersensitivity*	257	145,033	0.572 (0.506, 0.646)		Skin reaction	148	8,897	5.490 (4.665, 6.460)	
Type I hypersensitivity	155	2,142	32.595 (27.670, 38.398)		Rash maculo-papular	105	12,327	2.784 (2.297, 3.375)	
Anaphylactoid reaction	59	1,849	10.679 (8.238, 13.843)		Skin exfoliation*	98	53,942	0.588 (0.482, 0.717)	
Immune system disorder*	32	8,545	1.216 (0.860, 1.721)		Skin disorder	94	20,986	1.457 (1.189, 1.785)	
Type II hypersensitivity	18	46	208.099 (115.101, 376.236)		Rash erythematous	93	24,335	1.242 (1.013, 1.523)	
Type IV hypersensitivity reaction	14	1,593	2.870 (1.695, 4.857)		Skin fissures	82	10,178	2.631 (2.117, 3.271)	
Type III immune complex mediated reaction	14	259	18.496 (10.793, 31.696)		Rash pruritic*	66	31,123	0.687 (0.540, 0.875)	
Anaphylactoid shock	9	201	15.170 (7.774, 29.603)		Pruritus generalised	64	14,652	1.420 (1.111, 1.816)	
Cytokine storm	8	384	6.886 (3.418, 13.871)		Skin lesion*	58	16,369	1.151 (0.889, 1.489)	
					Rash papular*	48	14,256	1.093 (0.823, 1.452)	
					Skin ulcer*	41	16,202	0.821 (0.604, 1.115)	
					Rash macular*	38	20,864	0.590 (0.429, 0.811)	
					Toxic skin eruption	38	5,605	2.210 (1.606, 3.041)	
					Dermatitis allergic*	22	7,257	0.984 (0.647, 1.495)	
					Stevens-Johnson syndrome*	21	10,107	0.674 (0.439, 1.034)	
					Dermatitis exfoliative generalised	13	2,109	2.007 (1.163, 3.463)	
					Pruritus allergic*	4	821	1.584 (0.593, 4.231)	

ISA-ADEs, Immune system and skin and subcutaneous tissue related ADE; PT, preferred term; SOC, systematic organ classification; ROR, reports odds ratio *, not defined as a signal.

For oxaliplatin, there were 12 ADEs that were not defined as ISA-ADE signals: drug hypersensitivity, immune system disorder, urticaria, skin exfoliation, rash pruritic, skin lesion, skin ulcer, rash papular, rash macular, dermatitis allergic, Stevens-Johnson syndrome, and pruritus allergic. The other 22 ISA-ADEs were set as target ADEs for risk assessment.

### 3.2 Risk assessment of ISA-ADE after oxaliplatin combined with ICIs

**FIGURE 1:**
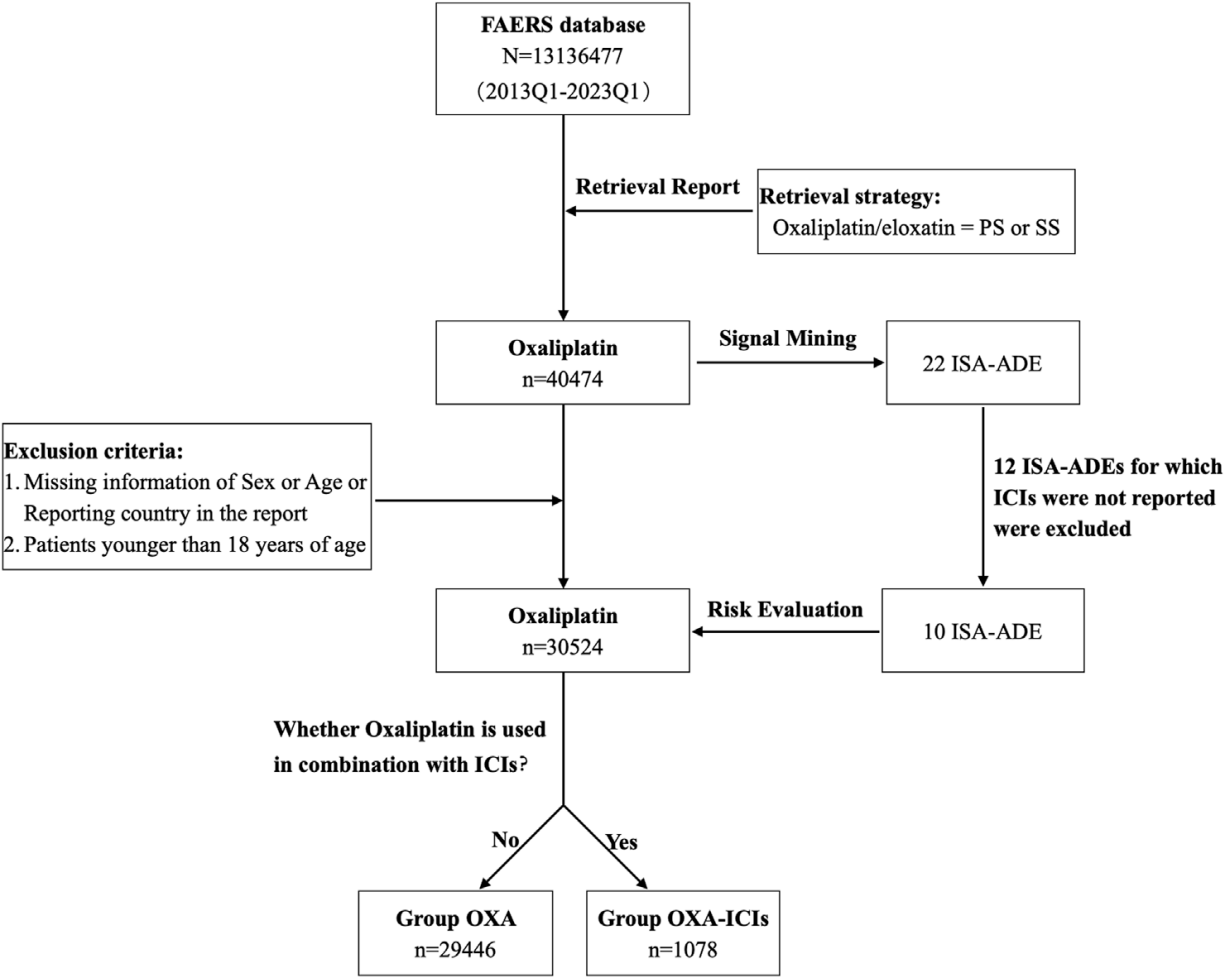
The flowchart of risk assessment of ISA-ADE after oxaliplatin combined with ICIs.

Of the 40,474 oxaliplatin reports retrieved, 30,524 patients were enrolled in the risk assessment study by applying the exclusion criteria. They were sorted into both Group OXA and Group OXA-ICI. The flowchart is shown in Figure 1. The characteristics of the patients in the two groups are listed in Table 3. There were significant differences in sex and age between the two groups (*p* < 0.01). In terms of the proportion of reports from each country (or region), France, Italy and Deutschland were the main countries in Europe, the United States was the main country in the Americas, and Japan and China were the main countries in Asia.

**TABLE 3:** Characteristic of 30,524 patients in Group OXA and Group OXA-ICI.

Characteristic	Group OXA (*n* = 29,446)	Group OXA-ICI (*n* = 1,078)	χ2 test *p*-value
Sex, No. (%)			<0.01
Female	12,785 (43.4)	345 (32.0)	
Male	16,661 (56.6)	733 (68.0)	
Age, mean (SD), y	61.5 (12.0)	62.2 (13.0)	<0.01
18–34, No. (%)	864 (2.9)	51 (4.7)	<0.01
35–64, No. (%)	15,225 (51.7)	482 (44.7)	<0.01
>65, No. (%)	13,357 (45.4)	545 (50.6)	<0.01
Country, No. (%)			
France	5,106 (17.3)	124 (11.5)	
Italy	4,095 (13.9)	18 (1.7)	
United States	3,935 (13.4)	165 (15.3)	
Japan	2,248 (7.6)	159 (14.7)	
Deutschland	2,148 (7.3)	199 (18.5)	
United Kingdom	1,769 (6.0)	28 (2.6)	
Netherlands	1,590 (5.4)	17 (1.6)	
China	1,579 (5.4)	78 (7.2)	
Spain	995 (3.8)	51 (4.7)	
Canada	869 (3.0)	28 (2.6)	
Other Countries	5,112 (17.4)	211 (19.6)	

Group OXA, group oxaliplatin; Group OXA-ICI, group oxaliplatin combined with Immune checkpoint inhibitor.

There were no reports of oxaliplatin in combination with cemiplimab, dostarlimab, or relatlimumab in the OXA-ICI group, so we did not perform a statistical analysis on these three drugs. When studying target ISA-ADEs, we found that 12 ISA-ADEs did not have oxaliplatin combined with ICI in the reports (see details in Supplementary Material S1), so only 10 ISA-ADEs were finally included in the risk assessment. See details in Table 4. We also found that no use of atezolizumab, durvalumab, or tremelimumab was reported in these 10 ISA-ADEs, so no statistical analysis was performed on these three drugs. See details in Supplementary Material S2.

**TABLE 4:** The number of ISA-ADEs in Group OXA and Group OXA-ICI and the value of odds ratios (10 PTs).

Preferred term (n)	Group OXA (*n* = 29,446)	Group OXA-ICI (*n* = 1,078)	Crude odds ratio point estimate (95% CI)
Hypersensitivity (*n* = 774)	760	14	0.497 (0.292, 0.846)
Anaphylactic reaction (*n* = 302)	295	7	0.646 (0.304, 1.370)
Cytokine release syndrome (*n* = 171)	165	6	0.993 (0.439, 2.248)
Anaphylactic shock (*n* = 244)	239	5	0.569 (0.234, 1.384)
Rash (*n* = 763)	713	50	1.960 (1.462, 2.628)
Pruritus (*n* = 685)	683	2	0.078 (0.020, 0.314)
Skin toxicity (*n* = 270)	269	1	0.101 (0.014, 0.718)
Rash maculo-papular (*n* = 92)	89	3	0.921 (0.291, 2.913)
Skin disorder (*n* = 69)	66	3	1.242 (0.390, 3.957)
Rash erythematous (*n* = 83)	81	2	0.674 (0.165, 2.744)

ISA-ADE, Immune system and skin and subcutaneous tissue related ADE; PT, preferred term; Group OXA, group oxaliplatin; Group OXA-ICI, group oxaliplatin combined with Immune checkpoint inhibitor.

In the risk assessment of each ISA-ADE between the two groups, only rash showed an increased risk in the OXA-ICI group, with an OR_cr_ of 1.96. See details in Table 4. We further adjusted the OR by sex and age, yielding an OR_adj_ of 1.974 (95% CI = 1.472, 2.647). We also conducted a further risk assessment for each ICI in combination with oxaliplatin. In combination of nivolumab with oxaliplatin, the OR_cr_ was 2.196 and the OR_adj_ was 2.231, while in the combination of pembrolizumab with Oxaliplatin, the OR_cr_ was 2.762, and the OR_adj_ was 2.678. See details in Table 5.

**TABLE 5:** The value of ORs of Rash between Group OXA and Group OXA-ICI.

Group	Total (N = 30,524)	Rash (N = 763)		
		n	Crude odds ratio point estimate (95% CI)	Adjust odds ratio # point estimate (95% CI)
Oxaliplatin	29,446	713	Reference	
Oxaliplatin-ICIs	1,078	50	1.960 (1.462, 2.628)	1.974 (1.472, 2.647)
-Nivolumab	653	33	2.196 (1.543, 3.125)	2.231 (1.567, 3.176)
-Nivolumab + Ipilimumab	70	1	0.565 (0.078, 4.071)	—
-Pembrolizumab	245	16	2.762 (1.655, 4.609)	2.678 (1.604, 4.471)

Group OXA, group oxaliplatin; Group OXA-ICI, group oxaliplatin combined with Immune checkpoint inhibitor #, Adjusted by Sex and Age.

## 4 Discussion

Oxaliplatin is a third-generation platinum compound that differs from cisplatin and carboplatin, there has been a low incidence of hypersensitivity reactions caused by oxaliplatin in early studies (Thomas et al., 2003; Shepherd, 2003). The signs and symptoms of oxaliplatin-induced hypersensitivity reactions are broad, frequently difficult to define, and include all organ systems (Shepherd, 2003). The mechanism of oxaliplatin-induced hypersensitivity is not fully understood, though most studies agree that it is a type I hypersensitivity reaction (Thomas et al., 2003). Oxaliplatin may act as a superantigen to cause cell proliferation and activation and then release cytokines (IL-6 or TNF-α). Another possible mechanism is the combination of oxaliplatin and major histocompatibility complexes to mediate the immune response (Thomas et al., 2003; Newman Taylor et al., 1999).

In this study, we focused on reports of ADEs of hypersensitivity reactions in the immune system and skin tissues. These ADEs are well known, despite the large number of pharmacovigilance signals in the classification of skin and subcutaneous tissue disorders. In contrast, little attention has been paid to pharmacovigilance signals under the classification of immune system disorders. Among them, type II hypersensitivity was a typical pharmacovigilance signal. Type II hypersensitivity reactions are primarily driven by IgG and IgM antibodies, the most common promotion mechanism being opsonization of antigen-bearing cells with antibodies, followed by phagocytosis or destruction. This can occur via two mechanisms: antibody-dependent cellular cytotoxicity (ADCC) and classical (antibody-mediated) complement activation (Knol and Gilles, 2022). Therefore, our findings contradict the conclusion proposed by some studies that suggest a link between oxaliplatin allergy and type I hypersensitivity reaction (Thomas et al., 2003; Newman Taylor et al., 1999). This finding deserves attention and should be known by both clinicians and patients. Other researchers have studied the occurrence of this ADE, including in patients within a chemotherapy cycle and at a specific time, but our study is limited by not having the report forms themselves and cannot confirm the information mentioned in the above studies.

The conventional chemotherapy drug oxaliplatin in combination with ICIs has been widely used in specific solid tumours, and we therefore performed an additional risk assessment based on previous studies of oxaliplatin signal mining. The major study was to investigate whether the combination of oxaliplatin and ICIs increases the risk of ISA-ADE in patients. The results showed that patients who used ICIs had a roughly two-fold increased risk of developing rash compared with those who did not. Among the ICIs used in combination with oxaliplatin, nivolumab and pembrolizumab also showed an increased risk of rash, at 2.196 and 2.231 times, respectively. These are all new discoveries. Considering that the patient’s sex and age may be risk factors for such ADEs (Parel et al., 2014; Kim et al., 2012), we also adjusted for these two factors, but combination therapy still increased the risk of rash. At the same time, there was a negative correlation between age and increased risk (see details in Supplementary Material S3), meaning that the likelihood of such an increase in risk decreased with patient age. This is also an interesting result.

In platinum-based chemotherapy regimens, a preparation of 5-fluorouracil, epidermal growth factor receptor tyrosine kinase inhibitor (EGFR-TKI) or vascular endothelial growth factor receptor tyrosine kinase inhibitor (VEGFR-TKI) is commonly used simultaneously, and these drugs can also cause ISA-ADEs (Oyama et al., 2021; Moret et al., 2022). In our study, however, we first qualified the identity of the target drug, that is, explained that it had to be a PS or SS in the report, thus ensuring the role of oxaliplatin in the occurrence of each ISA-ADE while reducing the effect of other drugs on the outcome. ISA-ADEs are among the most common irAEs noted in patients treated with ICIs. However, there is insufficient clinical evidence to suggest an increased risk of ISA-ADEs when ICIs are combined with certain drugs. While the current study found that ICIs in combination with oxaliplatin increased the risk of oxaliplatin-induced rash, there was no statistically significant increase in risk for different ISA-ADEs, which may be related to the fact that oxaliplatin in combination with ICIs has few clinical indications. Moreover, with the exception of nivolumab and pembrolizumab, ICIs were the least common drugs combined with oxaliplatin, which could be related to the late initiation and fewer ADE reports for other drugs.

In any case, as far as the interpretations of this study’s results are concerned, certain limitations should be considered: the incompleteness of the contents in the spontaneous reports involving missing data, and substantial bias may occur because of the spontaneous and voluntary reporting of ADEs. Although RORs for adverse events can be calculated from the data, they are only an estimate of the actual incidence of adverse events (Stephenson and Hauben, 2007). In addition, this study involved a statistical analysis of data within a certain period, which could not possibly include all reports of adverse events. Therefore, the results may be at a risk of overestimating the occurrence of the adverse events. This study focused on the possible association between the PS or SS drug and ADEs, and there were still confounding factors that may affect the results (such as tumor type, other drugs used in combination, etc.).

## 5 Conclusion

To our knowledge, there are still a few ADEs associated with immune system disorders induced by oxaliplatin that have not received enough attention, particularly type II hypersensitivity, which showed strong intensity signals as a pharmacovigilance signal. Due to the lack of research comparing the occurrence and related influencing factors of ISA-ADEs when oxaliplatin is used in combination with ICIs, the results of this study are a strong evidence supplement. We observed an approximate 2-fold increase in the risk of rash when oxaliplatin was combined with ICIs. ICIs used in combination with oxaliplatin, nivolumab and pembrolizumab have also been shown to increase the risk of rashes. And there was a negative correlation between age and increased risk. Our research has some inherent limitations due to its research nature, so it is necessary to further observational real-world studies are warranted to understand the occurrence of ISA-ADEs when oxaliplatin and ICIs are used in combination, and to optimize clinical practice.

## Data Availability

The original contributions presented in the study are included in the article/Supplementary Material, further inquiries can be directed to the corresponding author.
