# Supplementary material for: Risk of immune system and skin and subcutaneous tissue related adverse events associated with oxaliplatin combined with immune checkpoint inhibitors: a pharmacovigilance study
Source: Front Pharmacol. 2024 Jun 14;15:1309540. doi: 10.3389/fphar.2024.1309540 (PMC11211528; doi:10.3389/fphar.2024.1309540)
Supplement: Supplementary file 1 [file Table1.DOCX]

**Supplementary table S1 The number of ISA-ADEs was in Group OXA and Group OXA-ICI**

| **Preferred Term** | **Hypersensitivity**  **(n=774)** | **Anaphylactic reaction**  **(n=302)** | **Cytokine release syndrome**  **(n=171)** | **Anaphylactic shock**  **(n=244)** | **Type I hypersensitivity**  **(n=74)** | **Anaphylactoid reaction**  **(n=46)** | **Type II hypersensitivity**  **(n=11)** |
| --- | --- | --- | --- | --- | --- | --- | --- |
| **Group** |  |  |  |  |  |  |  |
| **Oxaliplatin (n=29446)** | **760** | **295** | **165** | **239** | **74** | **46** | **11** |
| **Oxaliplatin-ICI (n=1078)** | **14** | **7** | **6** | **5** | **0** | **0** | **0** |
| -Nivolumab | **11** | **7** | **3** | **5** |  |  |  |
| -Ipilimumab |  |  | **1** |  |  |  |  |
| -Nivolumab + Ipilimumab | **1** |  |  |  |  |  |  |
| -Pembrolizumab |  |  | **2** |  |  |  |  |
| -Atezolizumab |  |  |  |  |  |  |  |
| -Durvalumab |  |  |  |  |  |  |  |
| -Tremelimumab |  |  |  |  |  |  |  |
| -Avelumab | **2** |  |  |  |  |  |  |

ISA-ADE, Immune system and skin and subcutaneous tissue related ADE; Group OXA, group oxaliplatin; Group OXA-ICIs, group oxaliplatin combined with Immune checkpoint inhibitors

**Continue**

| **Preferred Term** | **Type IV hypersensitivity reaction**  **(n=13)** | **Type III immune complex mediated reaction**  **(n=11)** | **Anaphylactoid shock**  **(n=8)** | **Cytokine storm (n=7)** | **Rash**  **(n=763)** | **Pruritus**  **(n=685)** | **Skin toxicity**  **(n=270)** |
| --- | --- | --- | --- | --- | --- | --- | --- |
| **Group** |  |  |  |  |  |  |  |
| **Oxaliplatin (n=29446)** | **13** | **11** | **8** | **7** | **713** | **683** | **269** |
| **Oxaliplatin-ICI (n=1078)** | **0** | **0** | **0** | **0** | **50** | **2** | **1** |
| -Nivolumab |  |  |  |  | **33** | **1** | **1** |
| -Ipilimumab |  |  |  |  |  |  |  |
| -Nivolumab + Ipilimumab |  |  |  |  | **1** | **1** |  |
| -Pembrolizumab |  |  |  |  | **16** |  |  |
| -Atezolizumab |  |  |  |  |  |  |  |
| -Durvalumab |  |  |  |  |  |  |  |
| -Tremelimumab |  |  |  |  |  |  |  |
| -Avelumab |  |  |  |  |  |  |  |

ISA-ADE, Immune system and skin and subcutaneous tissue related ADE; Group OXA, group oxaliplatin; Group OXA-ICIs, group oxaliplatin combined with Immune checkpoint inhibitors

**Continue**

| **Preferred Term** | **Skin reaction**  **(n=106)** | **Rash maculo-papular**  **(n=92)** | **Skin disorder**  **(n=69)** | **Rash erythematous (n=83)** | **Skin fissures**  **(n=71)** | **Pruritus generalised**  **(n=58)** | **Toxic skin eruption**  **(n=18)** |
| --- | --- | --- | --- | --- | --- | --- | --- |
| **Group** |  |  |  |  |  |  |  |
| **Oxaliplatin (n=29446)** | **106** | **89** | **66** | **81** | **71** | **58** | **18** |
| **Oxaliplatin-ICI (n=1078)** | **0** | **3** | **3** | **2** | **0** | **0** | **0** |
| -Nivolumab |  | 1 | 2 | 2 |  |  |  |
| -Ipilimumab |  | 1 |  |  |  |  |  |
| -Nivolumab + Ipilimumab |  |  |  |  |  |  |  |
| -Pembrolizumab |  |  | 1 |  |  |  |  |
| -Atezolizumab |  |  |  |  |  |  |  |
| -Durvalumab |  |  |  |  |  |  |  |
| -Tremelimumab |  |  |  |  |  |  |  |
| -Avelumab |  | 1 |  |  |  |  |  |

ISA-ADE, Immune system and skin and subcutaneous tissue related ADE; Group OXA, group oxaliplatin; Group OXA-ICIs, group oxaliplatin combined with Immune checkpoint inhibitors

**Continue**

| **Preferred Term** | **Dermatitis exfoliative generalized**  **(n=12)** |
| --- | --- |
| **Group** |  |
| **Oxaliplatin (n=29446)** | **12** |
| **Oxaliplatin-ICI (n=1078)** | **0** |
| -Nivolumab |  |
| -Ipilimumab |  |
| -Nivolumab + Ipilimumab |  |
| -Pembrolizumab |  |
| -Atezolizumab |  |
| -Durvalumab |  |
| -Tremelimumab |  |
| -Avelumab |  |

ISA-ADE, Immune system and skin and subcutaneous tissue related ADE; Group OXA, group oxaliplatin; Group OXA-ICIs, group oxaliplatin combined with Immune checkpoint inhibitors
