# Supplementary material for: Risk of immune system and skin and subcutaneous tissue related adverse events associated with oxaliplatin combined with immune checkpoint inhibitors: a pharmacovigilance study
Source: Front Pharmacol. 2024 Jun 14;15:1309540. doi: 10.3389/fphar.2024.1309540 (PMC11211528; doi:10.3389/fphar.2024.1309540)
Supplement: Supplementary file 2 [file Table2.DOCX]

**Supplementary Table S2 The number of ISA-ADEs in Group OXA and Group OXA-ICI and the value of Odds Ratios**

| **Group** | **Total**  **(N=30524)** | **Hypersensitivity**  **(N=774)** | | **Anaphylactic reaction**  **(N=302)** | | **Cytokine release syndrome (N=171)** | | **Anaphylactic shock**  **(N=244)** | |
| --- | --- | --- | --- | --- | --- | --- | --- | --- | --- |
|  |  | **n** | **Crude Odds Ratio**  **Point Estimate (95% CI)** | **n** | **Crude Odds Ratio**  **Point Estimate (95% CI)** | **n** | **Crude Odds Ratio**  **Point Estimate (95% CI)** | **n** | **Crude Odds Ratio**  **Point Estimate (95% CI)** |
| **Oxaliplatin** | **29446** | 760 | - | 295 | **-** | 165 | **-** | 239 | **-** |
| **Oxaliplatin-ICI** | **1078** | 14 | 0.497 (0.292, 0.846) | 7 | 0.646 (0.304, 1.370) | 6 | 0.993 (0.439, 2.248) | 5 | 0.569 (0.234, 1.384) |
| -Nivolumab | **653** | 11 | 0.715 (0.402, 1.272) | 7 | 1.086 (0.511, 2.308) | 3 | 0.816 (0.260, 2.562) | 5 | 0.957 (0.393, 2.327) |
| -Ipilimumab | **93** |  |  |  |  | 1 | 1.935 (0.268, 13.962) |  |  |
| -Nivolumab + Ipilimumab | **70** | 1 | 0.556 (0.077, 4.012) |  |  |  |  |  |  |
| -Pembrolizumab | **245** |  |  |  |  | 2 | 1.466 (0.362, 5.945) |  |  |
| -Avelumab | **133** | 2 | 0.586 (0.145, 2.371) |  |  |  |  |  |  |

ISA-ADE, Immune system and skin and subcutaneous tissue related ADE; Group OXA, group oxaliplatin; Group OXA-ICI, group oxaliplatin combined with Immune checkpoint inhibitors

**Continue**

| **Group** | **Total**  **(N=30524)** | **Rash**  **(N=763)** | | **Pruritus**  **(N=685)** | | **Skin toxicity**  **(N=270)** | | **Rash maculo-papular**  **(N=92)** | |  |
| --- | --- | --- | --- | --- | --- | --- | --- | --- | --- | --- |
|  |  | **n** | **Crude Odds Ratio**  **Point Estimate (95% CI)** | **n** | **Crude Odds Ratio**  **Point Estimate (95% CI)** | **n** | **Crude Odds Ratio**  **Point Estimate (95% CI)** | **n** | **Crude Odds Ratio**  **Point Estimate (95% CI)** | |
| **Oxaliplatin** | **29446** | 713 | **-** | 683 | **-** | 269 | **-** | 89 | **-** | |
| **Oxaliplatin-ICI** | **1078** | 50 | 1.960 (1.462, 2.628) * | 2 | 0.078 (0.020, 0.314) | 1 | 0.101 (0.014, 0.718) | 3 | 0.921 (0.291, 2.913) | |
| -Nivolumab | **653** | 33 | 2.196 (1.543, 3.125) * | 1 | 0.065 (0.009, 0.466) | 1 | 0.169 (0.024, 1.204) | 1 | 0.502 (0.070, 3.607) | |
| -Ipilimumab | **93** |  |  |  |  |  |  | 1 | 3.624 (0.500, 26.282) | |
| -Nivolumab + Ipilimumab | **70** | 1 | 0.565 (0.078, 4.071) | 1 | 0.631 (0.087, 4.548) |  |  |  |  | |
| -Pembrolizumab | **245** | 16 | 2.762 (1.655, 4.609) * |  |  |  |  |  |  | |
| -Avelumab | **133** |  |  |  |  |  |  | 1 | 2.522 (0.349, 18.235) | |

ISA-ADE, Immune system and skin and subcutaneous tissue related ADE; Group OXA, group oxaliplatin; Group OXA-ICI, group oxaliplatin combined with Immune checkpoint inhibitors

**Continue**

| **Group** | **Total**  **(N=30524)** | **Skin disorder**  **(N=69)** | | **Rash erythematous**  **(N=83)** | |
| --- | --- | --- | --- | --- | --- |
|  |  | **n** | **Crude Odds Ratio**  **Point Estimate (95% CI)** | **n** | **Crude Odds Ratio**  **Point Estimate (95% CI)** |
| **Oxaliplatin** | **29446** | 66 | **-** | 81 | **-** |
| **Oxaliplatin-ICI** | **1078** | 3 | 1.242 (0.390, 3.957) | 2 | 0.674 (0.165, 2.744) |
| -Nivolumab | **653** | 2 | 1.367 (0.334, 5.590) | 2 | 1.130 (0.277, 4.605) |
| -Ipilimumab | **93** |  |  |  |  |
| -Nivolumab + Ipilimumab | **70** |  |  |  |  |
| -Pembrolizumab | **245** | 1 | 1.821 (0.252, 13.166) |  |  |
| -Avelumab | **133** |  |  |  |  |

ISA-ADE, Immune system and skin and subcutaneous tissue related ADE; Group OXA, group oxaliplatin; Group OXA-ICI, group oxaliplatin combined with Immune checkpoint inhibitors
