# Supplementary material for: Risk of immune system and skin and subcutaneous tissue related adverse events associated with oxaliplatin combined with immune checkpoint inhibitors: a pharmacovigilance study
Source: Front Pharmacol. 2024 Jun 14;15:1309540. doi: 10.3389/fphar.2024.1309540 (PMC11211528; doi:10.3389/fphar.2024.1309540)
Supplement: Supplementary file 3 [file Table3.DOCX]

**Supplementary Table S3 The results of binary logistic regression**

| **Item** | **Regression coefficient** | **Standard Error** | ***z* value** | **χ^2^** | ***p* value** | **OR** |
| --- | --- | --- | --- | --- | --- | --- |
| Age | -0.011 | 0.003 | -3.743 | 14.008 | 0.000 | 0.989 |
| Sex | 0.005 | 0.074 | 0.068 | 0.005 | 0.946 | 1.005 |
| ICIs | -1.310 | 0.805 | -1.626 | 2.644 | 0.104 | 0.270 |
| nivolumab | 2.202 | 0.818 | 2.692 | 7.246 | 0.007 | 9.045 |
| ipilimumab | -10.001 | 304.237 | -0.033 | 0.001 | 0.974 | 0.000 |
| nivolumab + ipilimumab | 7.545 | 304.240 | 0.025 | 0.001 | 0.980 | 1891.792 |
| pembrolizumab | 2.308 | 0.823 | 2.804 | 7.865 | 0.005 | 10.052 |
